# Advances in catalyst and reactor design for methanol steam reforming and PEMFC applications

**DOI:** 10.1039/d4sc06526c

**Published:** 2025-01-24

**Authors:** Eleana Harkou, Hui Wang, George Manos, Achilleas Constantinou, Junwang Tang

**Affiliations:** a Department of Chemical Engineering, Cyprus University of Technology 57 Corner of Athinon and Anexartisias 3036 Limassol Cyprus a.konstantinou@cut.ac.cy; b College of Environmental Science and Engineering, Hunan University Changsha 410082 P. R. China; c Department of Chemical Engineering, University College London (UCL) WC1E 7JE London UK; d Industrial Catalysis Centre, Department of Chemical Engineering, Tsinghua University Beijing 100084 China jwtang@tsinghua.edu.cn; e Ordos Laboratory Inner Mongolia 017000 China

## Abstract

Hydrogen (H_2_) is a clean energy carrier with significant potential for power and heat generation, offering a pathway to reduce emissions from fossil fuels. Over the years, various feedstocks have been explored for H_2_ production, addressing the storage challenges associated with hydrogen. Methanol (MeOH) has emerged as one of the most efficient hydrogen storage medium. Among the different MeOH conversion processes, steam reforming stands out for its high hydrogen selectivity. This review focuses on recent catalyst development, in particular MSR reactor design and configuration, an area that has received comparatively limited attention in previous studies. Innovative reactor configurations, such as membrane and small-scale reactors, address the limitations of traditional packed-bed units, including pressure drop, heat and mass transfer resistances, and scalability challenges. By systematically analysing various reactor configurations, we address a critical gap in existing reviews and deliver innovative strategies for process optimisation. Additionally, the integration of methanol steam reforming with fuel cell systems presents a promising solution for reducing emissions in the transport sector. The review also discusses the relevant understanding on reaction mechanisms involved, followed by both the challenges and future prospects, emphasizing the importance of evaluating not only the environmental impact of these emerging technologies but also their manufacturing and operational costs.

## Abstract 2

The review focuses on recent developments in methanol steam reforming for hydrogen production, highlighting innovative reactor designs and catalyst advances and applications in fuel cells, as well as the environmental and economic implications.

## Graphical abstract

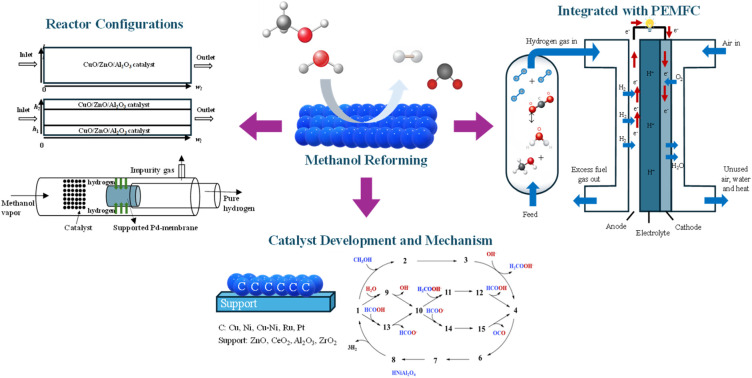

## 1. Introduction

The escalating environmental crisis, driven primarily by human activities and the extensive exploitation of fossil fuels for transportation, energy, and industrial purposes, necessitates urgent transitions to sustainable energy solutions. Hydrogen (H_2_) has emerged as one of the most promising alternatives, offering a clean energy carrier capable of replacing fossil fuels through its conversion into power or heat.^1^ The application of H_2_ in fuel cell technology demonstrates comparable performance to traditional fossil-fuel-based systems, making it an attractive option for various applications, including vehicles and heating systems. However, challenges related to its production cost, storage and transportation, persist, hindering the widespread adoption of H_2_ technologies.^2^

Integrating H_2_ into energy strategies is both crucial and necessary, particularly in the context of decarbonization efforts within the transportation and industrial sectors.^3^ One of the primary challenges associated with H_2_ is its low volumetric energy density, which complicates storage and increases costs. To address these issues, various storage methods have been explored, including liquid hydrogen, compressed hydrogen, and chemical storage using materials such as ammonia, metal hydrates, synthetic hydrocarbons, carbohydrates, formic acid, and liquid organic hydrogen carriers (LOHCs).^4,5^ Among these, methanol (MeOH) has garnered significant attention as an effective liquid H_2_ carrier due to its high H_2_ conversion efficiency.^6^ MeOH is a preferred H_2_ carrier as it also has the same H/C ratio as methane and is a liquid under standard environmental conditions. Additionally, its conversion to H_2_ occurs at lower temperatures, requiring less energy than fuels with C–C bonds, and it produces lower levels of CO due to milder operating conditions.^7^

Methanol Steam Reforming (MSR) stands out as a mature and economically feasible technology for H_2_ production. Industrially, MeOH is converted to H_2_ through reforming, with steam reforming being the most established process. Among the various reforming methods—MSR, methanol partial oxidation (POX), and methanol autothermal reforming (ATR), MSR is preferred due to its better H_2_ to CO ratio.^8^ Furthermore, other H_2_ carriers such as formic acid, ammonia and hydrous hydrazine also show significant promise. Formic acid can be decomposed into H_2_ under mild conditions.^9^ In this liquid organic hydrogen carrier (LOHC) system, CO_2_ is utilized in the formation of formic acid, making it a safer and more convenient H_2_ carrier to handle.^10^ Ammonia is also regarded as an excellent H_2_ carrier molecule as it possesses a high H_2_ content of around 18 wt% and a large energy density of up to 3000 W h kg^−1^. Hydrous hydrazine, with a high H_2_ content of 8 wt%, can also be decomposed to produce H_2_ and nitrogen (N_2_) by a carbon-free process. However, an undesired side reaction can generate ammonia, which affects the selectivity towards H_2_ and the overall efficiency of the process.^11,12^

Despite the extensive research on catalyst development for methanol reforming, the studies have primarily concentrated on catalyst-related aspects, with less attention given to innovative reactor technologies and whole chemical processes.^8,13–19^ This review aims to fill this gap by providing an up-to-date overview of various reactor units used in MSR, detailing their operational parameters and highlighting the benefits of membrane and microreactor systems besides a discussion on new catalyst development and mechanistic results. Additionally, this review examines the integration of the MSR reaction with Proton Exchange Membrane Fuel Cells (PEMFCs), extending beyond catalyst-related topics to include mechanistic insights.

## 2. Thermostatic materials

Due to the rise of environmental issues, the evaluation of existing energy processes and operations is a crucial factor concerning the research community. Among the products of the reaction, small amounts of CO are required for PEMFC applications. Emphasis should be placed on catalyst design to achieve high selectivity toward H_2_, making the produced CO negligible.^13^ The two side reactions that occur during the MSR are the MeOH decomposition and the water gas shift (WGS) reaction, which occurs when the steam/MeOH ratio is low. In contrast to MeOH POX, MSR is an endothermic reaction that yields high H_2_ and is widely preferred.^14^

In recent years, efforts have been made to improve the performance of conventional catalysts regarding the MSR reaction since Cu-based catalysts suffer from poor stability, sintering and carbon deposition, with the latter two being the most frequent issues. Improving preparation methods, adding promoters and optimising support materials could significantly enhance the stability of such catalysts.^20^ Moreover, the introduction of nanoparticles or porous materials can further improve conventional catalysts.^21^ Many catalysts have been suggested for H_2_ production showing long-term stability under high temperature conditions over a suitable support. Table 1 summarises the key features regarding the investigated catalysts.

**Table 1:** Thermostatic materials investigated for MSR

Catalyst	Pressure (bar)	Temperature (°C)	S/C ratio	MeOH conversion (%)	CO selectivity (%)	H_2_ selectivity (%)	H_2_ yield (mol/mol_MeOH_)	Ref.
10% Ni–Cu/Al_2_O_3_	—	250	2	100.0		—	2.66	22
Ni_0.2_–Cu_0.8_/ZrO_2_	1	325	—	—		—	2.00	23
Cu–Ni/TiO_2_/monolith	1	300	2	92.6	9.6	92.7	—	24
CuZrAl_0.4_	1	270	1.5	96.0		—	—	25
CuZnZrAl(Co–Am)	1	270	1.2	100.0	0.3	—	—	26
ZrO_2_-0.1/Cu	1	300	1	90.0	—	—	—	27
Cu/ZnO/Al_2_O_3_–5Mg	1	200	1	68.0	—	—	—	28
CuCe–MC (IMP)	—	250	3	68.0	17.0	>95.0	—	29
5% Cu/CeO_2_	—	340	—	76.0	1.1	—	—	30
5% Cu/Sm_2_O_3_	—	340	—	76.0	0.9	—	—	30
2% Pt/α-MoC	60	190	—	—	<0.1	—	—	31
15Pt/15In_2_O_3_/CeO_2_	1	350	1.4	99.9	2.9	74	—	32

Noble metals, such as Rh and Pd, were found to be advantageous by achieving high H_2_ yields and good stability, although their high cost hinders their application to different commercial systems.^33^ Ni-based catalysts, due to their low cost, are preferred for the MSR reaction, and their combination with Cu can significantly reduce CO formation. In a recent study, a Ni–Cu/Al_2_O_3_ catalyst was investigated, showing that by increasing the Ni content and catalyst loading almost 100% MeOH conversion was achieved, but at the same time the increase of the Ni content enhanced the MSR performance and RWGS reaction leading to higher CO concentrations.^22^

**Fig. 1:**
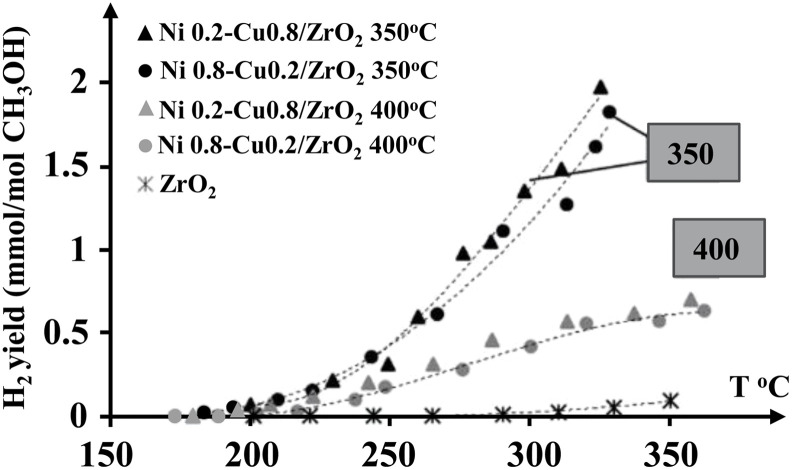
H_2_ yields of different ratios of Ni–Cu catalysts.^23^ Reproduced from ref. 23 with permission from Elsevier, Copyright 2015.

Bimetallic Ni–Cu catalysts supported on ZrO_2_ were examined by Lytkina *et al.*,^23^ with different ratios of nickel and copper and annealing temperature. Fig. 1 displays the H_2_ yields of the investigated catalysts with temperature gradient. Maximum H_2_ production was achieved at a lower annealing temperature (350 °C), where the sample was primarily represented by non-crystallised mass. MeOH-involved H_2_ production hardly occurred on pure Zr. Conversely, with calcined catalysts at 350 °C, a higher H_2_ yield could be obtained. However, the most active samples were the ones where the tetragonal ZrO_2_ was coated with an amorphous shell. Also, the conversion of MeOH on Cu-rich catalysts was more selective in the H_2_ production. The difference between the Cu-rich and Ni-rich catalysts in selectivity was due to the character of the predominant metal, showing itself in a change of Fermi level, as well as in chemical properties.

Tahay *et al.*^24^ studied the MeOH-involved H_2_ production process utilising a micro-structured monolith in conjunction with a synthesised nanostructure of TiO_2_. The surface of the monolith/TiO_2_ was coated with Cu, Cu/Ni, Ru and Pt metals. The monolith structure significantly improved the mass transfer of reactants and products, and, in synergy with the high surface area, porosity, pore size, and stability of the nanostructured TiO_2_ film, the MeOH conversion was found to reach 99% with low CO selectivity (5%). Among the investigated catalysts, the Ru catalyst exhibited the highest conversion and selectivity. However, due to economic considerations, the Cu–Ni catalyst presents an excellent alternative, achieving a 93% conversion rate. Furthermore, similar to previous studies, the MeOH conversion and H_2_ selectivity of the Cu–Ni catalyst were superior to those of the pure Cu catalyst, attributed to the role of Ni.

The primary challenge with commercial catalysts is deactivation, where various cocatalysts or promoters play a crucial role in enhancing the reforming stability and activity.^34^ For example, acidic or alkaline cocatalysts can further convert undesired products into valuable or less harmful ones, thereby improving the catalytic performance. Although highly dispersed Cu-based catalysts have been reported to achieve high activity with reduced CO formation, there is no consensus on the promotion mechanism.^35,36^ Moreover, the inclusion of a second promoter, along with diverse supports, may influence both activity and selectivity. There has been sustained interest in supports and promoters, with certain materials, such as ZnO, CeO_2_, Al_2_O_3_ and ZrO_2_, showing dual functionality by improving catalyst activity and stability through modifications in dispersion and interactions with the support material.^37^ It is widely recognized that isolated metal atoms exhibit exceptional performance in various reaction systems. However, the absence of ensemble sites limits their ability to facilitate the reaction of large molecules and certain multistep processes. Consequently, the “ensemble effects” between single metal atoms and neighbouring oxygen vacancies can enhance and promote the catalytic activity for reactions involving large molecules.^38^ The catalytic activity of Cu-based catalysts has been found to improve with both ZrO_2_ and ZnO promoters, which could enhance the surface area, stabilise Cu crystal size, prevent agglomeration of Cu particles during reduction and reaction processes, and stabilise Cu^+^ species on the catalyst surface.^39^ For instance, Pd/ZrO_2_ provided the best activity for H_2_ generation, but Pd/ZnO gives a high CO_2_ selectivity (97%).^40^ Different synthesis approaches impart distinct properties to catalytic materials. For instance, the sol–gel auto-combustion method used in the synthesis of Cu/ZrO_2_ catalysts has been found to achieve higher CO_2_ selectivity, activity, and stability compared to catalysts prepared through impregnation and co-precipitation methods.^41^

Mateos-Pedrero *et al.*^25^ investigated the performance of a newly synthesised catalyst, Cu over a ZrAl support. The new catalyst aims to improve the catalytic performance and promote non-CO generation. The major drawbacks of the most conventional Cu-based catalysts are the absence of lifelong stability and hence the activity loss and the formation of CO. The investigation showed that the composition of the support has a huge effect not only on the catalyst performance but also on the physicochemical characteristics of the CuZrAl catalyst. Moreover, the inclusion of zirconia and its content in the supported catalyst yielded interesting results. A high zirconia content led to lower activity due to zirconia segregation, as Zr has a low surface area. Conversely, at a low zirconia content, ZrAl samples exhibited a homogeneous composition with high dispersion of Zr and Al species. This improved dispersion and reducibility of the catalyst proved advantageous for catalytic performance.

Li *et al.*^26^ synthesised different CuZnZrAl catalysts *via* the coprecipitation-ammonia method as well as investigated the influence of Y and Ce promoters. The results revealed that this preparation method enhanced the dispersion of each component in the catalyst, leading to smaller crystal sizes and larger surface areas. The addition of promoters, particularly Ce, improved the catalyst's stability, achieving 100% conversion at 270 °C with a CO selectivity of just 0.3%. Furthermore, after 20 cycles, the conversion remained as high as 98%.

Xu *et al.*^27^ developed an inverse ZrO_2_/Cu catalyst through an oxalate sol–gel co-precipitation followed by calcination/H_2_-reduction treatment. The catalyst consisted of highly dispersed t-ZrO_2_ nanofragments with size between 3 and 4 nm over the Cu substrate with particles of around 20 nm size. The inverse catalyst showed exceptional stability in terms of long-term catalytic performance with zero CO production and a high yield of 190 mmol_H_2__ g_cat_^−1^ h^−1^ at 200 °C. Both DFT calculations and experimental results demonstrated that the highly reactive interface of –OH groups resulting from the formation of a ZrO(OH)–(Cu^+^/Cu) interfacial structure during the reaction can convert HCHO* to H_2_ and CO_2_ with HCOOH* as an intermediate.

Cheng *et al.*^28^ used the base of an industrial Cu/ZnO/Al_2_O_3_ catalyst to enhance the Cu–ZnO synergy. CuZnAl–*x*Mg catalysts were synthesised with different Mg loadings, and the addition of a Mg dopant was found to promote the catalytic activity, reaching the greatest space time yield of H_2_ of 172 mmol g^−1^ h^−1^ at 5% Mg content, attributed to the high surface area of Cu and abundant Cu^+^ species. Characterisation techniques revealed a decrease in the particle size and an increase in the surface area with higher Mg loadings. XRD patterns indicated that up to 5% Mg, the crystal size of ZnO and Cu was reduced, enhancing the catalytic performance. However, when the Mg composition was increased to 7%, the crystal size of ZnO and Cu enlarged, suggesting that the positive effect of Mg may be weakened in the presence of excess Mg.

Li *et al.*^42^ aimed to expose a commercial Cu/ZnO/Al_2_O_3_ catalyst to a mixture of H_2_, H_2_O, and CH_3_OH at atmospheric pressure and 300 °C to accelerate the migration of ZnO_*x*_ species onto the surface of Cu metal through an adsorbate-induced strong metal–support interaction. The results demonstrated that such morphological modifications could enhance the stability by approximately 70%. Moreover, the catalytic activity was improved, and the MSR reaction was promoted.

The influence of Ce and Zn on a Cu-based mesoporous carbon (MC) catalyst was studied by Bepari *et al.*^29^ These catalysts are widely used for their large pore volume, well-structured porosity, and chemical inertness. The addition of Cu to the MC increased the pore volume due to the formation of Cu nanoparticles on the support surface, while the addition of Zn and Ce further increased the pore diameter in nearly all catalysts. Catalytic testing, conducted between 200 and 350 °C using a wet impregnation method for CuCe on MC, demonstrated the best conversion rate among the catalysts, achieving 68% at 250 °C. In terms of conversion, the addition of Ce using both the one-pot and wet impregnation methods resulted in higher performance compared to CuZn on MC prepared by the one-pot method. H_2_ selectivity was high (>90%) for all catalysts, and the addition of Ce effectively inhibited carbon deposition.

**Fig. 2:**
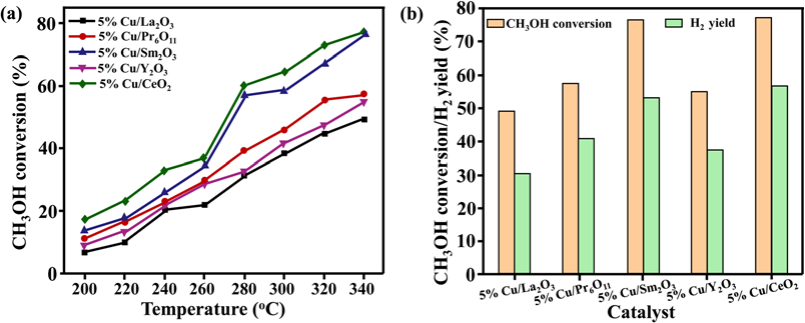
Catalytic MSR reaction tests. (a) MeOH conversion and (b) H_2_ yield at 340 °C.^30^ Reproduced from ref. 30 with permission from Elsevier, Copyright 2023 Elsevier Ltd. All rights reserved.

Liu *et al.* made an attempt to synthesise more feasible catalysts and for the first time investigated Cu-based catalysts on different supports, including La_2_O_3_, Pr_6_O_11_, Sm_2_O_3_, Y_2_O_3_ and CeO_2_.^30^ Five different rare earth oxide (REO) supports were used to study the Cu/CuO–support interaction, revealing that the La_2_O_3_ support had a negative impact on the reaction, while all the other cubic-phase catalysts were successfully synthesised. Regarding MSR reaction performance, among the five synthesised catalysts, the 5% Cu/CeO_2_ catalyst showed the best production performance, closely followed by the 5% Cu/Sm_2_O_3_ catalyst (Fig. 2). It was found that the quantities of surface basic sites, the active O_2_^−^ species, the Cu^+^ content/percentage and the active Cu surface area positively affected the reaction performance. Moreover, the MeOH adsorbing/SR *in situ* DRIFTS results revealed that in the case of Cu/CeO_2_ catalyst the m-HCOO* reactive monodentate intermediate was formed, while in the case of Cu/Y_2_O_3_ it was absent, making it the worst performing catalyst.

A Pd–Zn alloy was studied for thermocatalytic MeOH-involved H_2_ production, and it was found that the catalyst underwent pre-reduction before the reaction would facilitate the catalyst activity. The mechanistic investigation revealed that the formed HCHO underwent decomposition into CO and H_2_ on metallic Pd, whereas HCHO present on the Pd–Zn alloy was transformed into CO_2_ and H_2_ through the interaction with H_2_O.^43^ Additionally, Ru was reported to enhance the activity of thermocatalytic methanol-involved H_2_ production by promoting the dispersion of Pd particles. Characterisation results indicated that the promotion of CO desorption from Pd sites by Ru was the key factor for the observed activity enhancement.^44^ Moreover, Au served as an efficient cocatalyst in CeO_2_ catalysed MeOH-involved H_2_ production at low temperatures (<250 °C), where strong bonded Au–O–Ce species were the main active species.^45^ A single-atom system was also reported, in which Au and Pt were anchored on the lattice-O of ZnO.^46^ Density functional theory (DFT) calculations suggested that the efficient catalysis of Pt_1_ and Au_1_ originated from the strong binding energy of the intermediates, reducing the reaction barrier height and maximising active atom utilisation.

**Fig. 3:**
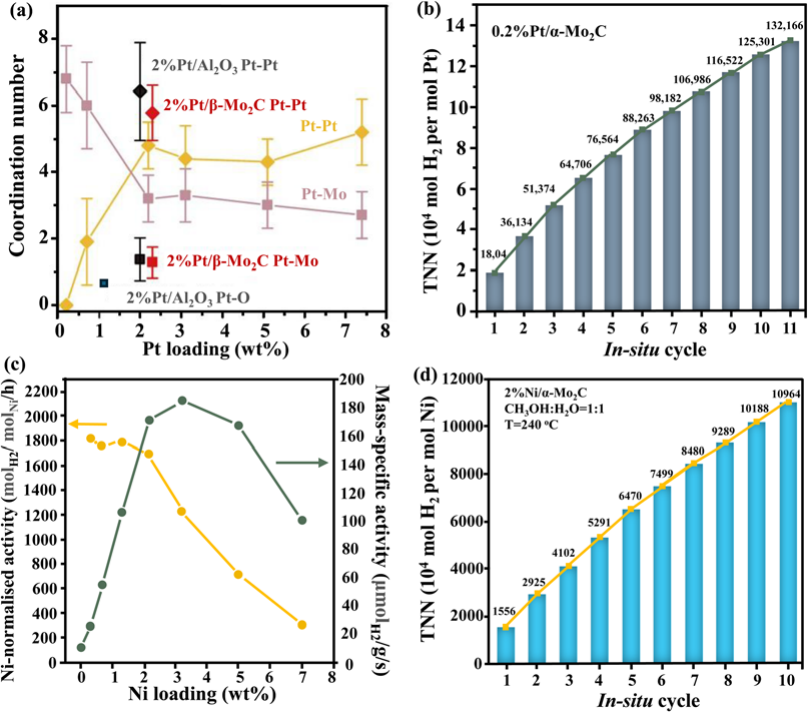
(a) Coordination numbers as a function of Pt loadings in Pt/α-MoC catalysts. (b) MeOH-involved H_2_ production activity on 0.2% Pt/α-MoC,^31^ (c) average catalytic activity of Ni/α-MoC with different Ni loadings and (d) MeOH-involved H_2_ production activity on 2% Ni/α-MoC.^47^ TTN: total turnover number. (a) and (b) Reproduced from ref. 31 with permission from Spring Nature, Copyright 2017. (c) and (d)Reproduced from ref. 47 with permission from American Chemical Society, Copyright 2021.

Lin *et al.*^31^ mentioned that Pt atomically dispersed on α-molybdenum carbide (α-MoC) enables low-temperature, base-free H_2_ generation, with remarkable H_2_ production activity in an aqueous phase system (Fig. 3a and b). Numerous studies have demonstrated H_2_ generation through methanol-involved aqueous phase reforming over Pt- and Ru-based noble metal catalysts. The exceptional H_2_ production was attributed to the strong ability of α-MoC to promote water dissociation and the synergistic effect between Pt and α-MoC, which effectively activated methanol and facilitated its reforming.

In a recent study of Lin *et al.*,^47^ the MeOH-involved H_2_ production was investigated using a newly discovered catalyst, Ni/α-MoC, where catalyst's production rate was 6 times greater than that of a conventional noble metal Pt/Al_2_O_3_ catalyst (Fig. 3c and d). The synergistic effect of CO reforming and C–H bond dissociation over the atomically dispersed Ni, as well as the highly efficient O–H activation over α-MoC, were the main reasons for effective catalytic performance. The key factor for highly active Ni-based catalysts relies on the high surface area with adequate Ni dispersion along with the small particle size.^48^

Modragón-Galicia *et al.*^49^ conducted a systematic investigation of the heterogeneous MSR reaction using three different catalytic materials: Pt/ZnO, Pd/ZnO, and PtPd/ZnO. X-ray diffraction (XRD) studies and profiles presenting crystallographic details revealed that the intermetallic PtZn phase on the Pt/ZnO catalyst was more stable compared to the PdZn phase on the Pd/ZnO catalyst. Additionally, platinum within the PtZn structure was found to be stabilized in the bimetallic PtPd/ZnO catalyst. Superior performance was demonstrated by measuring the catalytic reactivity of the Pt/ZnO-rod catalyst.

A Pt-based catalyst was investigated by Shanmugam *et al.*^32^ on the MSR. The study examined the influence of various metal supports (CeO_2_, Al_2_O_3_, and ZrO_2_) and the presence of In_2_O_3_ as a co-support, which yielded significant results. The addition of the co-support facilitated the formation of metallic Pt nanoparticles with high concentration, improved dispersion, and controlled particle size on the surface. The activity and stability of these catalysts were enhanced, with a notable reduction in CO formation. Among the catalysts, the Pt/In_2_O_3_/CeO_2_ catalyst exhibited excellent performance in MSR, demonstrating stability for 100 hours with the lowest CO formation. Additionally, single-atom Pt_1_ deposited on CeO_2_ offered significantly higher H_2_ activity, which was 40 times greater than that of 2.5 nm Pt/CeO_2_.^50^

Recently, low-temperature MeOH-involved H_2_ production was reported,^51^ where the synergy of Pt single atoms and Lewis pairs allowed porous CeO_2_ to realise efficient H_2_ generation at 120 °C, and a very low CO (0.027%) was observed. The catalyst design played a crucial role in the activation of both MeOH and H_2_O, resulting in high H_2_ production at lower temperatures – a challenging feat for single active-site catalysts. The construction of dual active-site catalysts suggests that a metal active site with high capability for methanol activation can exhibit low water dissociation, while water activation was promoted on reducible metal oxides through interaction with the O atom at a Lewis acidic centre, followed by transfer of one H atom to the adjacent Lewis basic centre. The Pt_1_/PN–CeO_2_ catalysts exhibited at 135 °C a H_2_ generation rate of 199 mol_H_2__ mol_Pt_^−1^ h^−1^, which was a lot higher than those of Pt/Al_2_O_3_ (2.6 mol_H_2__ mol_Pt_^−1^ h^−1^), Pt/TiO_2_ (3.8 mol_H_2__ mol_Pt_^−1^ h^−1^) and Pt/C (0.7 mol_H_2__ mol_Pt_^−1^ h^−1^) catalysts under the same conditions. The conventional metal-based catalysts utilised at the industrial level for the MSR reaction cannot operate at low temperatures such as 100–165 °C where the dual active site catalysts operate, since the temperature according to the Arrhenius expression 
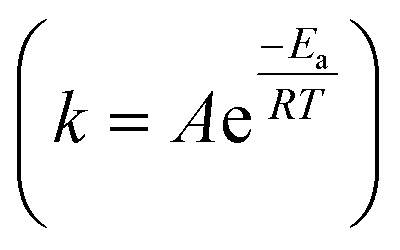
 is directly related to the reaction rate for H_2_ production. The development of such materials not only improves the energy efficiency of industries but also contributes positively to addressing the environmental crisis.

Zhang *et al.*^52^ conducted a morphological investigation evaluating different shapes of CeO_2_, including rods (r), cubes (c), and irregular (w) forms, on a Pd/In_2_O_3_/CeO_2_ catalyst prepared through the impregnation method. Catalytic performance results demonstrated that the Pd/In_2_O_3_/CeO_2_-r catalyst exhibited the best performance, achieving nearly 96% methanol (MeOH) conversion at 375 °C. Although the primary products were H_2_ and CO_2_, minor amounts of CO, methane, and dimethyl ether were also detected. CO selectivity was approximately 1.3%, while the selectivity of other undesired products remained very low, at parts-per-million (ppm) levels. Scanning Electron Microscopy (SEM) and High-Resolution Transmission Electron Microscopy (HR-TEM) characterization revealed that the shape of the CeO_2_ support affects the exposure of different crystalline surfaces, thereby influencing the catalytic activity. Specifically, in the rod-shaped morphology, the preferential exposure of the Ce (110) crystal facet provides abundant active Pd⁰ species, active oxygen vacancies, and surface oxygen. These features assist in the activation of MeOH and H_2_O during the catalytic cycle, thereby promoting the formation of CO_2_ and H_2_. Lastly, the stability of the Pd/In_2_O_3_/CeO_2_-r catalyst was highlighted, along with its potential for utilization in PEMFC systems.

Thermocatalytic MSR is a widely used industrial technology for H_2_ production, requiring high energy input due to the elevated operating temperatures. Efforts have been made to industrialise low-temperature MSR, attracting attention and finding applications in various areas. Research has primarily focused on catalyst development to improve the performance, selectivity, and stability. In addition to catalyst synthesis, other factors such as reactor design also influence the overall reaction performance and are discussed comprehensively below.

## 3. Reaction mechanism

The first attempt to explore the mechanism of surface reaction of different Cu-based catalysts was about three decades ago. The development of computational studies and hence the emphasis that has been given to the Cu-based surface reactions *via* DFT calculations are conducive to the better comprehension of the reaction mechanism while predicting catalyst surface pathways, reaction barriers and reaction energies for the MSR reaction.^53^ There are extensive studies on the reaction kinetics of Cu catalysts as well as noble metal catalysts,^54–56^ while recently we more often came across Ni-based catalyst investigations.

Bossola *et al.*^57^ designed a Cu-based catalyst on zirconia with the addition of silica to zirconia in order to improve the electronic and morphological properties of the Cu nanoparticles. A sequence of characterisation techniques were performed to address the improved activity, with the suggested mechanism proposing that MeOH is adsorbed on Cu, which then goes through the dehydrogenation process *via* the formate pathway. The metallic part of the nanoparticles contributes to the H_2_ production on the same site. Significant enhancement in the H_2_ production was obtained for Cu/ZrO_2_–SiO_2_, which was four times greater than that of the silica-free catalyst.

**Fig. 4:**
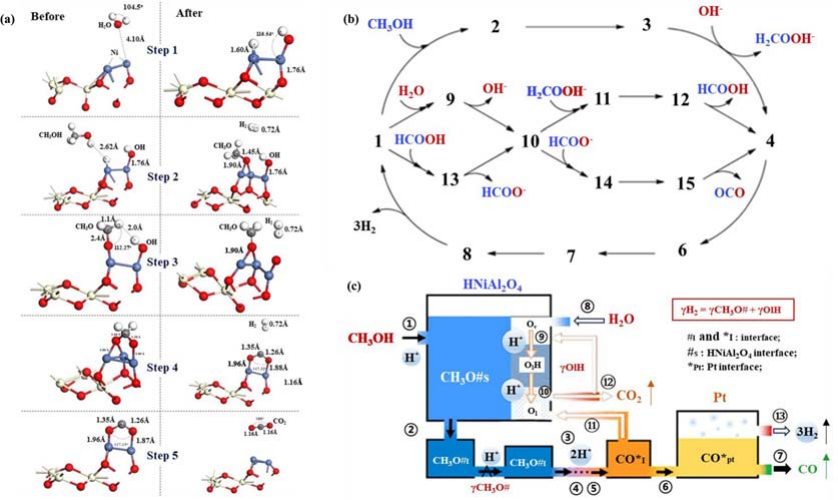
(a) Schematic of the suggested theoretical catalytic mechanism of CeO_2_ and Ni/CeO_2_ in MeOH-involved H_2_ production,^58^ (b) the ruthenium-catalysed production mechanism of H_2_ production from MeOH-involved H_2_ production,^62^ and (c) the overall process *via* Pt/NiAl_2_O_4_.^63^ (a) Reproduced from ref. 58 with permission from Elsevier, Copyright 2021 Elsevier B.V. All rights reserved. (b) Reproduced from ref. 62 with permission from American Chemical Society, Copyright 2014. (c) Reproduced from ref. 63 with permission from American Chemical Society, Copyright 2023.

Reyna-Alvarato *et al.*^58^ theoretically studied the MSR reaction mechanism using different catalysts, such as CeO_2_ and Ni/CeO_2_, showing similar results as Zuo *et al.*^59^ Their theoretical mechanism suggests that the reaction consists of 5 steps (Fig. 4a). Initially, the H_2_O molecule is adsorbed over the catalyst surface which forms one hydroxyl group (OH) and one adsorbed hydrogen (H), and MeOH then reacts with the atomic H_2_ forming a methoxy species (–CH_3_O) at the catalytic surface. In the third step, another molecule of H_2_ is released, forming one formaldehyde molecule (–CH_2_O), which is considered as the intermediate reaction. The new formate is a result of the reaction between the H_2_ molecule of the hydroxyl group and another H_2_ molecule of the methoxy group. Consequently, a H_2_ molecule is released with the subsequent decomposition of one formaldehyde molecule into H_2_ and O–C, where the former is surface attached. Lastly, a third H_2_ molecule and the remaining CO_2_ molecule are released.

Fajin and Cordeiro^60^ studied the MSR over Ni–Cu surfaces using DFT calculations to understand all the possible reaction routes, especially the ones achieving direct CO_2_ formation from MeOH. The decomposition on the surface followed by the WGS reaction, which directly transformed the obtained CO to CO_2_ and H_2_, the direct conversion of MeOH into CO_2_ and H_2_ and possible methane and coke formation are the catalytic reaction routes that were considered in their work. The disruption of the O–H bond or one of the C–H bonds of MeOH starts its decomposition with further rupture of the remaining bonds to follow. The direct formation of CO_2_ and H_2_, is a pathway affiliated to the presence of combined phases in the catalyst. Formaldehyde can be obtained during the MeOH decomposition reaction with a co-adsorbed hydroxyl, and the attained compound (CH_2_OOH) is converted on the surface until the generation of CO_2_. Furthermore, the catalyst blocks the generation of methane or coke, while the desorption of CO is not to be expected.

A single Ni-embedded model (NiCu (111)) and a single Ni-adsorbed model (A-NiCu (111)) were developed by Tang *et al.*,^61^ wherein DFT calculations were performed to assess their adsorption capacities and mechanistic investigation was carried out. Both the considered bimetallic surfaces were found to have enhanced stability compared to the pure Cu (111), and the introduction of Ni also improved the adsorption performance. Additional investigations were conducted on adsorption enhancement, showing that the A-NiCu (111) surface has a stronger adsorption promoting effect. Regarding the reaction mechanism, three major procedures were taken into account during the MSR (MeOH dehydrogenation, water decomposition and RWGS reaction), while several intermediate reactions were considered indicating that the rate-determined step is the methoxy dehydrogenation. Moreover, the lower activation energy of the NiCu (111) surface during the CO conversion process plays a significant role in inhibiting coke deposition.

The reaction mechanism of MSR using Ru-based catalysts was investigated by Yang *et al.*,^62^ wherein a DFT study was applied to reveal the catalytic cycles for the release of H_2_ and CO_2_. Three catalytic cycles participate in the reaction mechanism consisting of the MeOH to formaldehyde dehydrogenation, the formaldehyde coupling and the hydroxide to formic acid conversion as well as the formic acid dehydrogenation to CO_2_. Fig. 4b shows the overall mechanism of the MSR reaction. The reaction begins with a hydroxyl proton transfer from a molecule of MeOH to the ligand N_2_ of the catalyst and then the C–H bond splits with ease transferring the hydride to the metal centre of the catalyst for the formation of formaldehyde. The latter generates an anion that is a bit more stable with the hydroxide anion, which could be regarded as originating from the solvent or formed from the cleavage of H_2_O. Then, the formation of formic acid through the disruption of a C–H bond happens for the transfer of a methylene hydride in the H_2_COOH^−^ anion to Ru. Due to the acidity of formic acid, it is not possible to discover the transition state for the rupture of the O–H bond in formic acid. After every catalytic cycle, a molecule of H_2_ is released through a self-promoted mechanism that features an extra MeOH or H_2_O molecule acting as a bridge for the transfer of a proton from the ligand nitrogen to the metal hydride. Recently, steady-state isotopic transient kinetic analysis (SSITKA) was utilised to study the detailed process involving methoxyl and CO species adsorbed on Pt/NiAl_2_O_4_.^63^ From Fig. 4c, C–H bond cleavage occurs within methoxyl adsorbed on interface sites, and O–H bond rupture is observed within oxygen-filled surface vacancies, respectively.

Xue *et al.*^64^ aimed to investigate the gliding arc MSR (GA-MSR) reaction mechanism combining experimental data and plasma kinetic simulations at different MeOH concentrations. First, the H, OH and CH_3_ were considered as the main reactive species as their concentrations increased due to their fast formation in the inside arc stage, while they were consumed up in the outside arc step during the thermochemical process. The main steps considered were the MeOH decomposition and dehydrogenation. The increase of MeOH concentration demonstrated an enhancement solely on the concentrations of H and CH_3_, suggesting that at low MeOH concentrations CO_2_ is produced and at higher MeOH concentrations hydrocarbons are produced.

Almithn and Alhulaybi^65^ performed a mechanistic investigation using DFT calculations for Ni_2_P. It was revealed that compared to other transition metals such as Pt, Pd and Co that convert MeOH to CO, on Ni_2_P, the MSR reaction may compete with MeOH decomposition. The presented mechanism suggests that formaldehyde (CH_2_O*) reacts with the co-adsorbed OH* to produce CH_2_OOH*, which is then dehydrogenated to form CO_2_. It was also reported that the exceptional selectivity and the coke formation resistance of Ni_2_P could potentially be considered as a possible alternative solution for Cu-based catalysts.

## 4. Main experimental setups and reactors

The reactor configurations play a vital role in the performance of the MSR reaction and more specifically to the conversion of the reaction. The conventional reactor unit that is more frequently utilised for the MeOH-involved H_2_ production in thermocatalysis is the packed bed reactor. However, during recent years, efforts were made to develop other reactor designs such as membrane reactors (MR) and micro-reactors to enhance the H_2_ production.^66^ The smaller reactor units require better design deployment compared to the conventional units to avoid large pressure drops.^67^ According to Iulianelli *et al.*,^68^ most of the time, the MSR reactors and MR are tubular in order to compete with the better performance of more complex designs that have higher manufacturing costs. Table 2 summarises different reactor configurations utilised for MSR.

**Table 2:** Reactor set-ups utilised for the MSR

Reactor	Design characteristics	Conditions	MeOH conversion (%)	H_2_ yield (%)	Ref.
Continuous flow	Isothermal tubular packed bed reactor with an internal diameter of 1 mm and a length of 16 cm	230 °C, 0.86 bar, S/C = 1.1, and W/F = 35 kg_s_ g^−1^ mol^−1^	100.0	—	76
Continuous flow	Isothermal tubular coated wall reactor with an internal diameter of 4.1 mm and a length of 16 cm	230 °C, 0.86 bar, S/C = 1.1, and W/F = 40 kg_s_ g^−1^ mol^−1^	94.0	—	76
Continuous flow	Isothermal packed bed reactor with an internal diameter of 1.5 mm and a catalyst size of 150 μm	230 °C, 1 bar, S/C = 1.1, and *m*_cat_/*V*_in_ = 2 [mg (μL^−1^ min^−1^)]	60.0	—	78
Continuous flow	Isothermal coated wall reactor with an internal diameter of 1.5 mm a and catalyst size of 150 μm	230 °C, 1 bar, S/C = 1.1, and *m*_cat_/*V*_in_ = 2 [mg (μL^−1^ min^−1^)]	65.0	—	78
Continuous flow	Counter-flow tubular packed bed reactor with 8 internal heating tubes with an internal diameter of 12 mm and a length of 25 mm	*T*_in_ = 240 °C, 1 bar, and 8 heating tubes	94.5	61.7	79
Continuous flow	Tubular fixed-bed reactor with simultaneous internal and external heating	*T*_in_ = 250 °C, 1 bar, S/C = 1.5, and *v* = 0.2 m s^−1^	100.0	62.8	80
Continuous flow	Non-isothermal multitubular packed bed reactor with a length of 0.5 m and thermal air passing through the shell side	*T*_thermal air_ = 400 °C, *T*_MeOH_ = 160 °C, and 1 bar	95.0	65.0	82
Membrane	Packed bed reactor with Pd membrane for a H_2_ purity of >99.99%	280 °C, 1 bar, S/C = 1.2, and *Q* = 1 g min^−1^	97.0	91.1	83
Membrane	Fixed bed reactor with Pd membrane for a H_2_ purity of >99%	280 °C, 1 bar, S/C = 1.2, and *V* = 0.5 mL min^−1^	70.3	74.4	84
Membrane	Isothermal membrane reactor with a silica membrane (4 mm thickness and 5 cm active length)	300 °C, 1.5 bar, S/C = 1, and GHSV = 6000 h^−1^	85.0	85.0	85
Membrane	Isothermal membrane reactor with a Pd–Ag membrane (50 μm thickness and 5 cm active length)	280 °C, 2 bar, S/C = 1, and GHSV = 1800 h^−1^	100.0	100.0	85
Membrane	Isothermal membrane reactor with a silica membrane of 4 mm thickness	240 °C, 10 bar, S/C = 3, and GHSV = 6000 h^−1^	95.0	96.0	86
Membrane	Non-isothermal membrane reactor with 4 membranes of 3 in. height and 1/8 in. diameter dead-end tubes coated with a 30 μm thick Pd–Ag active layer	450 °C, 6 bar, S/C = 1, and *V* = 0.02 mL min^−1^	98.0	—	87
Membrane	Non-isothermal membrane reactor with a Pd–Ag membrane consisting of 600 ceramic support tubes	300 °C, 2 bar, S/C = 1, and sweep ratio = 1	94.0	93.8	88
Microreactor	Non-isothermal circle-triangle cross-sectional microreactor	*T*_in_ = 100 °C, 10 bar, and S/C = 1.2	98.6	—	89
Microreactor	Isothermal multichannel microreactor with 16 parallel mini channels	275 °C, 1 bar, S/C = 1.3, and WHSV = 0.67 h^−1^	89.7	—	90
Microreactor	Non-isothermal microreactor with parallel microchannels and the catalyst coated on the reactor's wall	500 °C, 1 bar, S/C = 10, and GHSV = 24 000 mL (g^−1^ h^−1^)	97.0	—	91
Microreactor	Isothermal microreactor based on 5 stacked wave sheets and copper foam	280 °C, 1 bar, and S/C = 1.5	65.0	—	92
Microreactor	Non-isothermal multilevel series scaled-up microreactor with 5 parallel MeOH combustion chambers	320 °C, 1 bar, and S/C = 1.3	92.5	—	93
Microreactor	Microreactor with a Ti porous membrane of 25 mm diameter and 1 mm thickness	360 °C, 1 bar, S/C = 8.8, and WHSV = 9.28 h^−1^	63.0	—	94

### 4.1. Packed bed reactors

A packed bed reactor loaded with solid catalytic particles is considered a simple system with low cost of manufacturing and operation. At the industrial level, they are preferred due to their easier design and construction, but at the same time there is a high pressure drop over the reactor.^69^ However, proper optimisation of operating conditions of packed bed reactors can significantly improve the H_2_ efficiency at the outlet. There have been many studies, recently, focusing of the packed bed reactor units conducting either experimental or theoretical investigations.^70–75^

Karim *et al.*^76^ compared an isothermal packed bed reactor and a coated-wall reactor, where the catalyst bed is a layer in contact with the wall of the reactor. Generally, the benefits of this reactor configuration vary. Some of the advantages are low-pressure drop, better heat and mass transfer and the lowest amount of catalyst to operate.^77^ In the packed bed reactor, it was found that between 1 and 4.1 mm internal diameter the heat transfer is limited and temperature gradients of up to 40 K could exist in the reactor bed. In contrast, the coated-wall reactor was free from any heat or mass limitations with lower pressure drop over the reactor and higher catalytic activity. The fact that MeOH can be reformed at low temperatures between 200 and 250 °C makes it even more attractive for large-scale applications.^76^

**Fig. 5:**
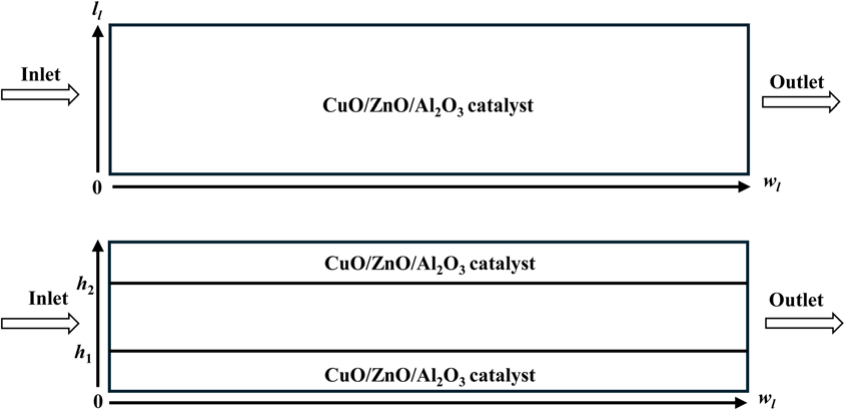
Schematic representation of the reforming reactor: (a) packed bed and (b) coated-wall packed bed microreactors.^78^ Reproduced from ref. 78 with permission from the Royal Society of Chemistry.

Hafeez *et al.*^78^ carried out a similar theoretical study comparing the isothermal packed bed and coated wall microreactors using a CuO/ZnO/Al_2_O_3_ catalyst (BASF F3-01), which is shown in Fig. 5a and b. Both microreactors appeared to have the similar performance at same temperatures due to the smaller scale of reformers. The coated wall reactor was expected to obtain higher conversion rates at a constant wall temperature in each layer, but the difference between the results of the two reactors was negligible due to the small size of reactors. The test regarding the robustness of the model by a comparison between 2D and 3D modelling configurations confirmed the model validity. Case studies such as the effect of residence time, temperature, steam to MeOH ratio, and thickness of catalyst coating on MeOH conversion were performed. More studies occurred, revealing that the bigger pellet sizes of catalyst led to the appearance of internal mass transfer resistance, followed by a reduction in MeOH conversion.

A numerical investigation was conducted by Kusumastuti *et al.*^79^ using a heating counter-flow tubular packed bed reactor. The tube design is extensively discussed in the literature for its straightforward construction. Three different configurations were assessed for their MSR reaction performance (straight tube, divergent tube and convergent tube), showing that the divergent design achieved the higher MeOH conversion (around 68%). Also, it highlighted the positive effect of internal heating with a waste heat source due to the endothermic reaction which dominates, enhancing the MeOH conversion. The number of heating tubes was another parameter that was investigated varying from 4 to 6 and to 8. The results demonstrated that a higher heating tube number tends to increase the conversion rate as improved heat transfer is accomplished. Additional studies were done to optimise the reactor design such as the position of the heating tube, the reformer angle and the inlet temperature, revealing that the best performance (almost 95% MeOH conversion) was obtained using the divergent shape with 8 heating tubes, a hot air inlet velocity of 0.5 m s^−1^, a heating tube position of 3.5 mm and a reformer angle of 5° with an inlet temperature of 513 K.

Another limitation that constitutes an obstacle for fuel cell applications and a frequent difficulty to be overcome in packed bed reactors is the irregular temperature distribution. A tubular fixed bed reactor with simultaneous external and internal heating was investigated by Zhang *et al.*^80^ to enhance the temperature distribution and hence the conversion of MeOH. A 3D validated model was utilised to simulate a tubular fixed bed reactor, a tubular fixed bed reactor with an inner heating pipe and a tubular fixed bed reactor with helical fins around the heating pipe. It reported the significant contribution of the heating pipe in the MeOH conversion and H_2_ production. Moreover, the addition of helical fins enhanced the convective heat transfer and flow path of reactants, achieving better performance of the reaction with an increase in the conversion of around 8.5%. It also examined the impact of geometric parameters, such as the pitch, height and width of helical fins, temperature, S/C molar ratio and inlet flow velocity, on the reaction performance. The optimum operating parameters showed almost 100% MeOH conversion.

A 3D numerical model of tubular packed bed reactor comprising several cylindrical tubes was utilised by Wang *et al.*^81^ for the MSR reaction. Effects of various parameters such as S/C molar ratio, inlet temperature, reactant mass flow rate, catalyst particle size and porosity were investigated for assessing the performance of the MSR reaction. The optimised conditions were determined as a temperature of 280 °C, S/C ratio of 2, inlet gas velocity of 0.065 m s^−1^ and catalyst particle size and porosity of 1 mm and 40%, respectively. The catalyst was located in different sections in order to avoid the formation of hot-spot areas inside the reactor in a large extent, especially in a segmented reactor configuration where the temperature inhomogeneity was reduced by almost 70% as well as the CO concentration by over 30%.

Zhu *et al.*^82^ examined the MSR reaction using a Cu/ZnO/Al_2_O_3_ catalyst in a non-isothermal multi-tubular packed-bed reactor with heating tubes operating as a heat exchanger as well. A comprehensive pseudo-homogeneous model was developed, and the validation was conducted following a previous study of Zhu *et al.*^95^ Two different flow set-ups have been investigated. A co-current design in which the reactant and air flow are in the same direction and a counter-current design where both flows are directed in opposite directions. The results demonstrated that both reactors showed high MeOH conversion over 95%, while the co-current design achieved the lowest CO concentration at the outlet of the reactor (0.34%). Additional investigations were carried out for the same catalyst mass by increasing the tube number while decreasing their diameter. An improvement in the MeOH conversion rate was obtained, but at the same time the amount of CO generated was increased.

In order to find applications, the MSR technology should be subject to an economic evaluation for its efficiency and feasibility. Choi and Stenger^96^ performed an economic evaluation of an optimized system with the optimized reactor size and operating conditions, using economic profit as the objective function. Here, profit is defined as the difference between H_2_ revenue and the fixed and operating costs of production. H_2_ revenue is affected by H_2_ purity, while reactor and energy costs are also important factors. MATLAB software was utilized for the analysis, where the profit function was maximized by minimizing the difference between cost and revenue through the optimized process.

A technoeconomic analysis by Rahatade and Mali^97^ was reported for the first time, considering the integration of heat and steam reforming processes of methanol (MeOH) and dimethyl ether (DME). A fixed-bed reactor was utilized in the simulations at a temperature of 250 °C and a pressure of 20 bar. The economic analysis was conducted by estimating the Total Annual Cost (TAC) of the process, which includes both the fixed capital investment (FCI) and working capital investment (WCI). The results indicated that the utilization of MeOH was cheaper than DME, as fewer utilities were involved. Moreover, heat integration was found to be more beneficial than a non-heat-integrated system, resulting in a reduction in TAC of up to $1610, while H_2_ production was approximately $10 300 cheaper than that from DME.

The majority of publications focused on kinetics, thermodynamics, modelling and simulation studies, as well as on evaluating the performance of different reactor designs. However, the technoeconomic analysis of different configurations for the MSR reaction is limited in the literature.

### 4.2. Membrane reactors

The recent development of PEMFCs as an alternative energy conversion technique aimed to eliminate greenhouse emissions and furthermore environmental pollution that originated from the conventional energy sources.^98^ This technology demands high clarity of H_2_, which can be achieved by membrane reactors (MRs). A membrane plays a vital role in the removal or addition of chemical species in a reaction system while also contributing to better interactions between the catalyst and reactants without conducting any separation process.^99^ These reactors can achieve higher conversions or the same conversion under milder conditions than conventional reactor systems. Moreover, high-purity H_2_ production is achieved in a sole unit. The high cost, low chemical resistance in the case of dense Pd-MRs, not high purity H_2_ production and perm-selectivity in the case of composite Pd-based MRs and the contamination of H_2_S and CO are some of the drawbacks of MRs.^100^

Since Pd-MRs are the most studied ones, Shi *et al.*^83^ developed a Pd membrane reaction system for pure H_2_ production. The double-functioned system allowed the low-temperature MSR reaction and the high-temperature purification conducted under isothermal conditions. Experimental results with optimisation revealed that high H_2_ purity (above 99.99%) was achieved for a MeOH flow rate between 1 and 2 g min^−1^ and pressure of 1.5–6 bar. Successful separation was achieved with ppm levels of CO concentration and furthermore showed potential to provide up to 500 W, finding application to power mobile devices and small range-extended vehicles.

**Fig. 6:**
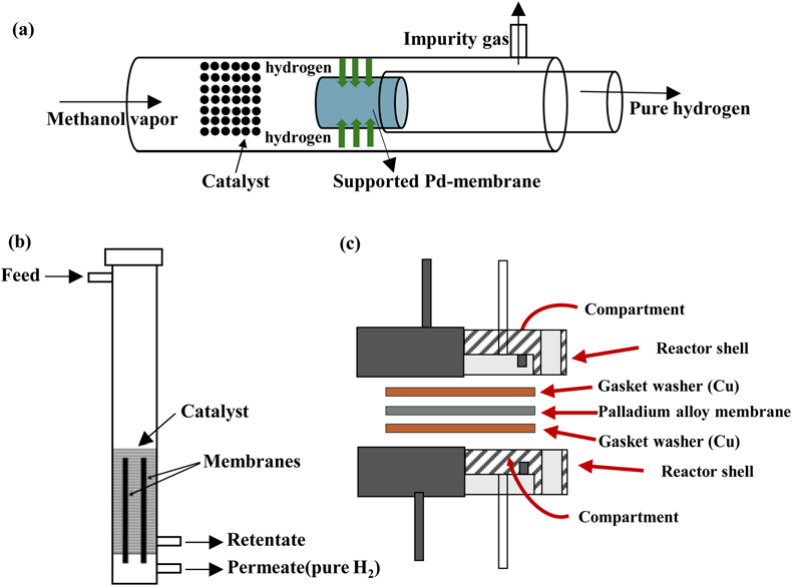
Schematic representations of MRs with the (a) ceramic supported Pd membrane,^84^ (b) Pd–Ag metallic active layer,^87^ and (c) Pd–Cu alloy membrane.^101^ (a) Reproduced from ref. 84 with permission from Elsevier, Copyright 2023. (b) Reproduced from ref. 87 with permission from Elsevier, Copyright 2022. (c) Reproduced from ref. 101 with permission from Pleiades Publishing Ltd, Copyright 2020.

A high H_2_ purification process was designed by Wang *et al.*^84^ for the production of pure H_2_ from the MSR reaction using the CuCe/Al_2_O_3_ catalyst and a Pd membrane supported on porous ceramic. Fig. 6a illustrates the schematic diagram of the H_2_ integrated MR. Characterisation techniques revealed the exceptional catalyst properties of high catalytic activity and stability. The H_2_-purification integrated reactor was operated at temperatures between 360 and 400 °C, showing an increase in the H_2_ generation, while MSR was conducted in a wide temperature range between 220 and 400 °C. At 400 °C the H_2_ concentration reached a maximum of almost 99.4%, and the CO concentration was found to be around 0.07 vol%. It was reported that the further increase of temperature over 400 °C, could lead to intermetallic diffusion between the Pd membrane and stainless-steel support, thereby decreasing the H_2_ permeance. In this study, the integrated reactor showed an exceptional stability performance for over 720 min with a high H_2_ concentration of above 99%.

Ghasemzadeh *et al.*^85^ investigated and evaluated the application of silica and Pd–Ag MRs in the MSR reaction. The CFD results show that both MRs have rather better performance than the conventional reactor, with the silica MR being the best choice. Also, it is evident that there are lower CO levels at the reactor effluent for the silica membrane as it is more permeable to H_2_, which emphasises the shift effect of the reaction, allowing higher MeOH conversion and a higher yield. Under isothermal conditions of 513 K and 5 bar, which was considered the optimum pressure for operation, the H_2_ yield was around 95%, while the temperature effect showed an increase in the H_2_ yield from 74 to 89% for temperatures between 493 and 573 K. Moreover, an increase in CO selectivity up to 2% was noted for the maximum operated temperature.

Further studies were performed by Ghasemzadeh *et al.*^86^ to assess the performance of different silica isothermal MR configurations. The examined configurations included a co-current flow design, a counter-current flow design and a counter-current flow design including the WGS reaction in the permeate side. All the investigated designs were found to outperform the conventional packed bed reactor. The H_2_ purity was one of the goals of this work, and it was analysed in the retentate and permeate streams of all the MR configurations. The counter-current flow design with the WGS reaction in the permeate side showed a better H_2_ permeation driving force compared to the other two configurations as well as a lower CO selectivity.

Cifuentes *et al.*^87^ studied the same reaction in a non-isothermal catalytic MR using Pd–Ag metallic membranes and a PdZn/ZnAl_2_O_4_/Al_2_O_3_ catalyst (Fig. 6b). The MR design was compared with a convectional packed bed reactor, whereas a 3D CFD non-isothermal model was designed and validated using experimental results including the mass transfer resistances. The experimental results demonstrated an exceptional H_2_ recovery of 85% at a temperature of 430 °C and an absolute pressure of 6 bar. Additionally, different operating conditions were evaluated to overcome the heat and mass transfer issues occurring in the reactor.

A study including a Ni–Cu catalyst was performed in a conventional reactor and MR by Mironova *et al.*^101^ It revealed that the H_2_ yield in the membrane is higher, and also the Pd–Cu alloy membrane exhibits high H_2_ permeability. The schematic representation of the MR is presented in Fig. 6c. Additionally, the membrane provides a high-purity H_2_ production (>97%), as a stream of pure H_2_ is collected in the permeate zone. An experimental investigation for the permeability of the membrane was conducted, showing that the increase of temperature significantly improved the H_2_ recovery rate up to 60%, while the heating from 300 to 400 °C showed great improvement in the H_2_ flux across the membrane. During the cooling period, the H_2_ flux across the membrane was found to be beyond that observed during the heating period for the same temperatures, attributed to the inhibition of phase transition from α to β upon modification of the Pd–Cu alloy, and thus the phase ratio varied with the increase or decrease of temperature.

Saidi^88^ evaluated the performance of a Pd–Ag catalytic membrane for pure H_2_ production using a commercial Cu/ZnO/Al_2_O_3_ catalyst. A 2D non-isothermal model was designed incorporating all the heat and mass transfer phenomena taking place in the MR, with the simulated results to be in a good agreement with the experimental ones. The effects of pressure, temperature, sweep ratio, and steam ratio on MeOH conversion and H_2_ recovery were also examined. The results revealed that the H_2_ recovery is enhanced with the temperature and pressure along the reactor length. The selective H_2_ removal shifts the reaction equilibrium towards the H_2_ formation, hence enhancing the conversion of the reaction. The model showed that at 2 bar, 573 K, and a sweep ratio of 1, the maximum H_2_ yield improved from 64% to 100% by increasing the steam ratio from 1 to 4.

The Pd membranes might be considered one of the most expensive membranes, but compared to other materials of lower cost, such as polymer membranes, they offer excellent H_2_ recovery and purity over 99% in most of the cases. At the same time, they suffer from embrittlement effects and low thermal and chemical resistances. However, their integration with metals could potentially improve their performance.^102^ MRs have a great opportunity for industrial applications as they offer a better solution for high MeOH conversion and high-purity H_2_ production compared to packed bed reactors as well as the simultaneous production and separation of reactants and products. Moreover, their lower mechanical and chemical stability and high cost could affect their industrialisation.

MRs are considered appropriate for MSR reaction systems as a plethora of investigations regarding the exceptional reactor's performance and H_2_ yield exist in the literature. Nevertheless, the focus of the most publications was to evaluate the performance of new catalytic materials and reactor designs as well as mechanistic and thermodynamic investigations. Therefore, the techno-economic analysis of the MSR reaction, studying the economic aspects of different scale systems, is limited.^103^ Byun *et al.*^104^ conducted a techno-economic analysis to assess the technical and economic feasibility of a packed bed reactor with membrane filters compared to a conventional membrane reactor. Aspen HYSYS software was utilized to investigate the impact of several techno-economic parameters. The analysis employed itemized cost estimation, where a unit's H_2_ production cost was calculated by dividing the total cost—defined as the sum of annualized capital cost ($ per year) and operating cost ($ per year)—by the total H_2_ production rate (kg per year). It was found that temperature had a significant influence on the reactor performance. The effect of H_2_ permeance showed similar results to the temperature effect, where lower unit H_2_ production costs could be obtained with higher H_2_ permeance. Moreover, it highlighted that a higher number of packed bed reactors and membrane filters do not necessarily assure cheaper H_2_ production costs at high temperatures where almost 100% of MeOH is converted.

A similar study was carried out by Kim *et al.*,^105^ who designed a reactor in Aspen HYSYS software to evaluate the technical and economic feasibility of an MR for ultra-pure H_2_ production. A unit H_2_ production cost was calculated by dividing the annual cost ($ per year) by the annual H_2_ production yield (kg H_2_ per year), comparing the MR with a conventional packed bed reactor based on process simulation results. The total annual costs for a packed bed reactor and the MR were $93 401 per year and $72 305 per year, respectively, with the capital cost of a membrane module being $5105. Furthermore, the capital expenditure (CAPEX) results demonstrated that CAPEX accounts for 3% in the MR, in contrast to 17% in the packed bed reactor, mainly due to the elimination of the pressure swing adsorption system in the MR. Itemized cost estimations revealed a unit H_2_ production cost of $9.37 per kg H_2_ for the packed bed reactor and $7.24 per kg H_2_ for the MR, respectively, showing approximately a 23% cost reduction in the MR.

### 4.3. Microreactors

The design of micro-reformers to convert hydrocarbon fuel to H_2_ has gained the interest of many scientists. Microchannel reactors provide a powerful tool for process intensification and microscale processing. These reactors are ideal for fast, high exothermic reactions, and they show superior heat and mass transfer rates.^106^ The structure and the design of microreactors affect their performance and hence the conversion of MeOH.^107,108^ However, despite their advantages, microreactors face challenges that limit their broader application. Their rigid structure reduces flexibility, making it difficult for them to adapt to dynamic environments or fit into limited spaces.^109^ Nevertheless, numerous studies in the literature highlight the exceptional performance of microreactors, which often surpasses that of conventional reaction units.^110–114^

Lu *et al.*^115^ investigated three types of microreactors to enhance the direct microchannel performance. Using numerical simulations, the results showed that the sinusoidal microchannel with dimples (SMD) is the optimal structure. In addition, the effects of reaction temperature, MeOH-feeding flux, and steam/MeOH mole ratio on microreactor's performance were examined, while different structures were investigated experimentally for the validation of the results. The SMD microreactor not only showed the best overall performance but also demonstrated significant improvements in heat and mass transfer, along with a high hydrogen production capacity.

A theoretical work focusing on the channel structures of microreactors was conducted by Liu *et al.*^89^ to address issues related to mass and heat transfer phenomena and improve the H_2_ yield. Five different channel structures were investigated, involving circle–triangle, circle–square, square–circle, square–square and triangle–circle designs (Table 3). Among these, the circle-triangle design exhibited the best performance, achieving methanol conversion rates above 98% and hydrogen selectivity exceeding 88%. The superior performance of the circle–triangle configuration was attributed to its larger coating area, which improved methanol conversion, and its enhanced its mass transfer rates, resulting from a more efficient velocity flow distribution compared to other channel designs.

**Table 3:** Five different channel structures.^89^ Reproduced from ref. 89 with permission from Elsevier, Copyright 2024

Channel type	Overall shape	Cross-sectional shape	Catalytic coatings	Combustion channel
Circle–triangle	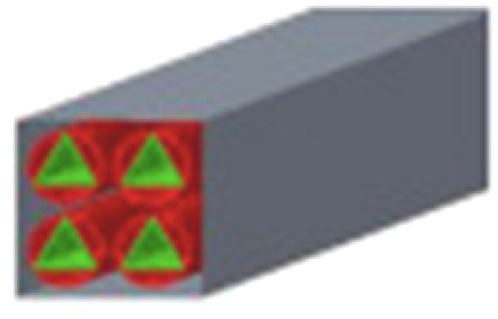	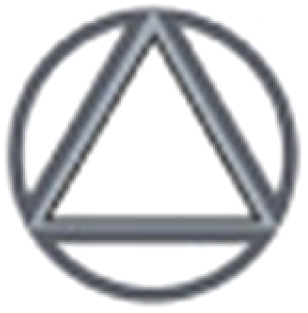	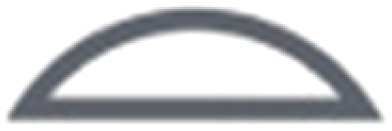	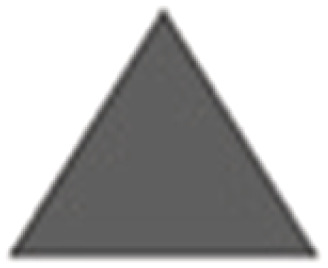
Circle–square	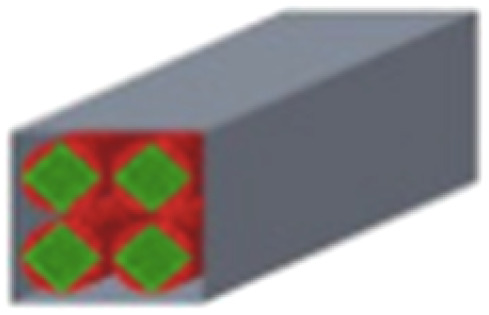	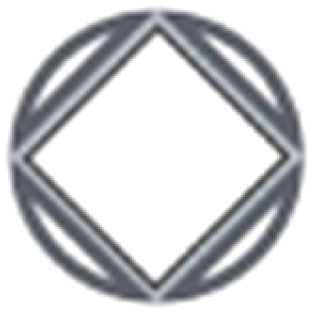	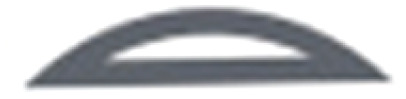	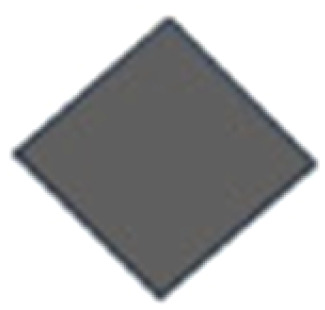
Triangle–circle	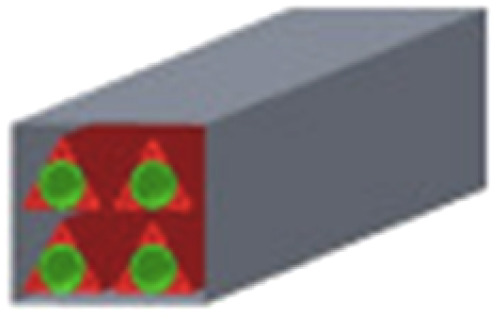	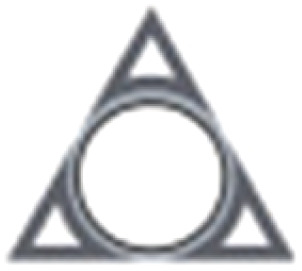	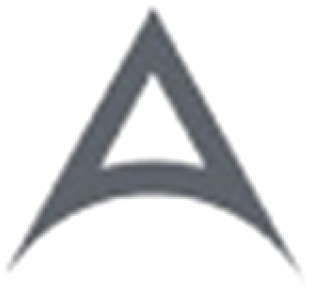	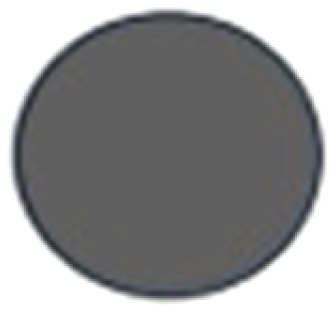
Square–circle	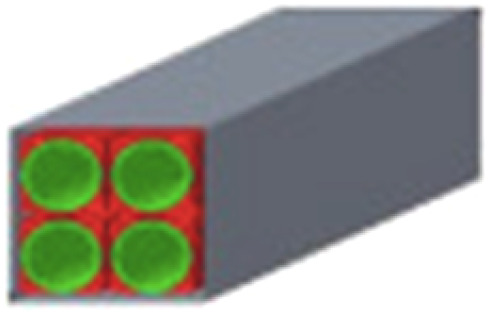	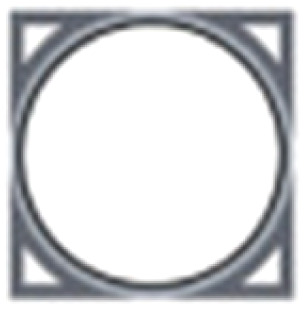	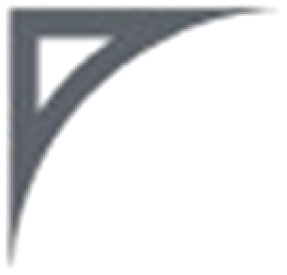	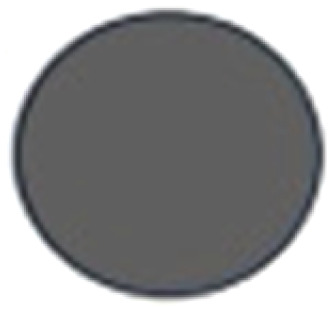
Square–square	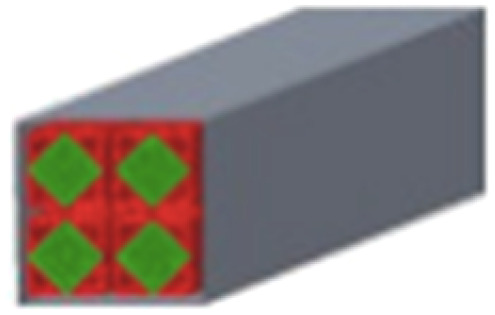	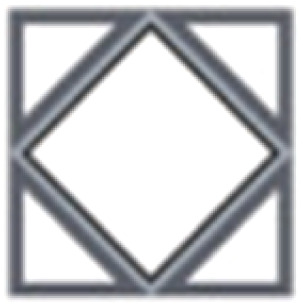	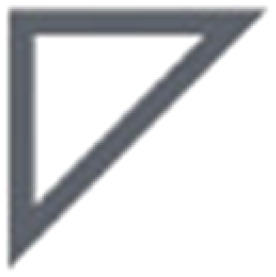	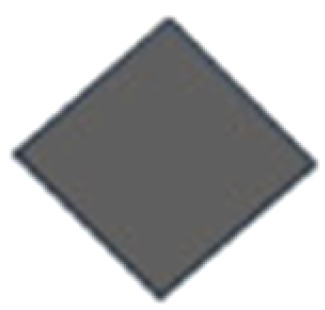

Zhuang *et al.*^90^ created a theoretical model to study the MSR reaction in an isothermal multichannel reactor. A commercial CuO/ZnO/Al_2_O_3_ catalyst was utilised in the investigation of sixteen parallel mini-channels. The 3D model accounted for heat and mass transfer within the reactor, providing accurate predictions for species consumption and generation, which were validated through experimental comparisons. The results showed minimal temperature differences (around 2.5 K), demonstrating uniform temperature and fluid velocity distribution across the sixteen parallel mini-channels, indicating the excellent performance of the micro-reactor.

Sarafraz *et al.*^91^ performed the MeOH-involved H_2_ production in a non-isothermal microreactor with parallel channels using a Cu–SiO_2_ catalyst coated on the wall surface. The experiments occurred at operating temperatures of 250–400 °C, reactant flow rates between 0.1 and 0.9 L min^−1^, catalyst loadings of 0.25–1.25 g and a heat flux value of 500 kW m^−2^. However, the reactor has an operating tolerance up to 600 °C. An increase of reactants’ gas hourly space velocity (GHSV) causes a reduction in conversion, which is due to the lower residence time, the suppression in diffusion of reactants into the pores of the catalyst and also the temperature of the average film. The MeOH conversion can reach 97% at 400 °C at a GHSV of 24 000 mol g^−1^ h^−1^ and a MeOH/water ratio of 1.

Wu *et al.*^92^ suggested an isothermal microreactor with stacked wave sheets and copper foam for highly efficient H_2_ generation from MSR. A fractal body-centred cubic model was used to study the flow characteristics and reaction performance of the copper foam with a coated catalyst layer. Experimental and theoretical results revealed that the reformate flow rate increased with the increase of the number of microreactor layers and the flow rate of MeOH. Both studies showed good agreement with only 7% differences in MeOH conversion. It was also observed that the stacked wave sheets and copper foam show a uniform reactant flow and improved H_2_ generation.

A scaled-up amplified non-isothermal microreactor was designed by Wu *et al.*^93^ to enhance the production of H_2_. Experimental and theoretical studies were conducted, where 5 MSR plates were used along with 5 MeOH combustion champers to improve the distribution of temperature. The velocity distribution within the microchannels and Cu foam showed better homogeneity in the first case. The microchannels and Cu foam combination resulted in better mixing of reactants. In addition, as the reforming chambers are uniformly designed, the flow velocity is symmetrically distributed. The results from MeOH combustion and MSR reaction showed that the microreactor was heated from 18.5 to 310 °C within 1618 seconds. Optimal operating conditions resulted in methanol conversion rates above 88%.

Fan *et al.*^94^ developed a membrane microreactor for the MeOH-involved H_2_ production using immobilised Cu/ZnO/Al_2_O_3_ nanoparticles in the pores of the membrane. Characterisation techniques verified that the nanoparticles were successfully immobilised. A comparison with a conventional packed bed reactor was conducted with the membrane microreactor to show an exceptional performance (5000 mmol h^−1^ H_2_ yield) which was one order of magnitude higher than that of the packed bed reactor. Additional investigations showed that there is no concentration gradient, and hence the velocity is uniformly distributed resulting in almost 100% H_2_ selectivity. It predicted that by stacking five membrane microreactor sheets, methanol conversion could exceed 95%.

A numerical modelling study of the MSR reaction in a heterogeneously catalysed microchannel SR reactor was conducted by Chen and Yu.^116^ The main goal of the study was to investigate the phenomena occurring within the microchannel reactor during the reactions. The effects of various parameters were evaluated to enhance the reactor's performance. The results indicated that mass transfer resistance was present despite the system's small scale, suggesting that design improvements are necessary to eliminate external mass transfer limitations. A maximum output power in excess of 70 W per channel was achieved, and energy efficiencies of up to approximately 70% were available. The operating limit line of MeOH breakthrough was found to influence the H_2_ yield, while an optimum velocity, along with adjustments to the flow rate and catalyst loading, was necessary to control and achieve high MeOH conversions.

Zhuang *et al.*^117^ experimentally investigated the performance of a novel isothermal multichannel micropacked bed reactor with a bifurcation inlet manifold and a rectangular outlet manifold. A commercial CuO/ZnO/Al_2_O_3_ catalyst was utilized to assess the effects of different parameters on MeOH conversion, H_2_ production, and CO concentration. The results demonstrated that temperature had a more significant impact on the reaction's performance compared to the S/C ratio and weight hourly space velocity (WHSV). Moreover, an improved MeOH conversion rate was obtained by increasing the S/C ratio and temperature while decreasing the WHSV and particle size. The reactor was suggested to be suitable for Proton Exchange Membrane Fuel Cell (PEMFC) applications for H_2_ production under operating conditions of an S/C ratio of 1.3, a temperature of 275 °C, a WHSV of 0.67 h^−1^, and a particle size between 150 and 200 mesh, achieving over 94% MeOH conversion and a CO yield of less than 1%.

Membrane reactors and microreactors present a promising alternative to conventional packed bed reactors for MSR, offering high hydrogen purity and conversion rates under milder conditions, as well as improved heat and mass transfer. However, cost remains a significant barrier for membrane reactors, despite their ability to integrate a reformer and a separator into a single unit, which could potentially lead to lower operating temperatures compared to conventional reactors. As noted by Kim *et al.*,^105^ comprehensive studies that combine both process simulation and economic analysis are scarce in the literature. Therefore, greater emphasis should be placed on the economic analysis of MRs to assess their economic feasibility. Furthermore, the MSR reaction operates at relatively low temperatures between 200 and 300 °C, as shown in Table 2, making it a competitive and attractive candidate for various applications. Additionally, strategies aimed at lowering the activation barrier of the reaction could further reduce operating temperatures, enhancing the viability of MSR systems. More research on scaling up microreactors is required to ensure that their advantages, such as uniform distribution, are maintained in industrial applications. Optimizing reactor designs for methanol reforming, especially for industrial uses, is crucial as current investigations are limited.

## 5. Integration of the MSR reaction and fuel cell systems

**Fig. 7:**
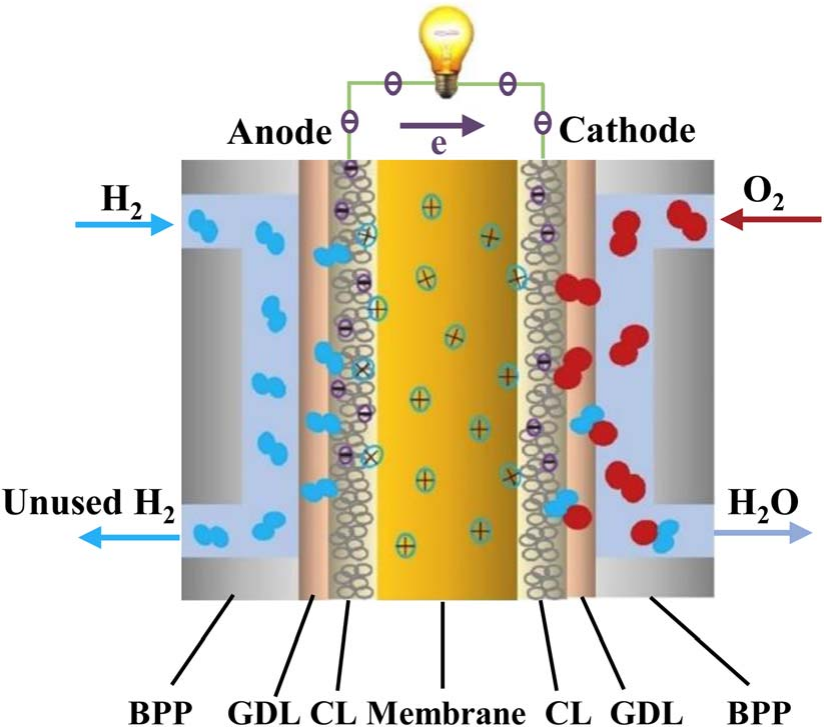
PEMFC schematic illustration.^121^ Reproduced from ref. 121 with permission from Elsevier, Copyright 2023.

Recent trends in the literature show that the fuel cell technology is a promising alternative for the elimination of emissions of commercial vehicles. Challenges that must be overcome are often ascribed to the high manufacturing cost. In order to compete the existing technologies and increase the demand, different strategic decisions must be considered by the manufacturing companies and the states.^118^ Fuel cell techniques can be separated into 5 types, the PEMFCs, alkaline fuel cells (AFCs), phosphoric acid fuel cells (PAFCs), molten carbonate fuel cells (MCFCs) and solid oxide fuel cells (SOFCs). Among the different kind of techniques, the most popular one is the PEMFCs.^119^ Ideally, membranes used in PEMFC systems should target the high conductivity of protons, low electronic conductivity, low fuel permeability, low drag coefficient of electroosmotic, good thermal/chemical stability and mechanical properties.^120^ The PEMFC system structure consists of bipolar plates, gas diffusion layers on both sides and a PEM in the middle of the configuration. Fig. 7 illustrates the proton formation and transportation from the anode to the cathode through the PEM to react with O_2_, producing electrons and hence water, electricity and heat.^121^

There are two categories of PEMFCs divided based on the temperature level of operation, low-temperature PEMFCs (LT-PEMFCs) and high-temperature PEMFCs (HT-PEMFCs), operating around 60–80 °C and 160–220 °C, respectively. Because of the low CO tolerance of LT-PEMFCs, this system faces poisoning issues at the catalyst anode simultaneously and thus the HT-PEMFCs are more advantageous,^122^ offering emission free conversions and simplified water and heat management and cooling systems. However, HT-PEMFCs due to their high operating temperatures carry safety risks and technical challenges and also incur high manufacturing costs.^123^ On the other hand, the reforming LT-PEMFC systems have high power density and proton conductivity as well as low heat loss, making it suitable for portable small-scaled devices.^124^ According to Ribeirinha *et al.*,^125^ the membrane composition plays a vital role in CO tolerance since polybenzimidazole (PBI) membranes and generally most of the PEMs can tolerate CO concentrations up to 3000 ppm and can be straightaway fed with MeOH stream without any purification process. It was also noted that the heat recovery is important during the operation to maximise the overall efficiency.^126^

**Fig. 8:**
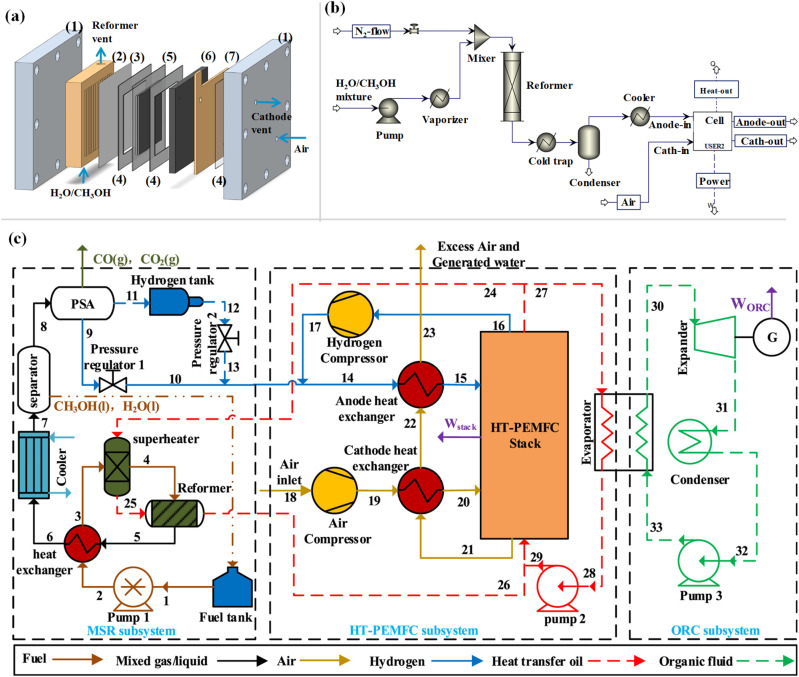
(a) Representation scheme of the cellular membrane packed bed reactor integrated with a HT-PEMFC: (1) metal end-frame plates, (2) gold coated reformer, (3) Pd–Ag membrane, (4) gasket, (5) MEA, (6) bipolar graphite plate and (7) current collector.^127^ (b) Flowsheet diagram of the integrated fuel cell system^130^ and (c) schematic diagram of the HT-PEMFC system integrated with an MSR reactor setup and an ORC subsystem.^131^ (a) Reproduced from ref. 127 with permission from Elsevier, Copyright 2018, (b) reproduced from ref. 130 with permission from Elsevier, Copyright 2023, and (c) reproduced from ref. 131 with permission from Elsevier, Copyright 2022.

An experimental and a theoretical investigation were performed by Ribeirinha *et al.*,^127^ using an integrated HT-PEMFC with a cellular membrane packed bed reactor. Fig. 8a illustrates the integrated system showing all the constituent parts. The Pd–Ag membrane on the reaction unit was employed, showing H_2_ permeability to suspend the poisoning of the anode by MeOH. However, the Pd–Ag membrane deactivation due to CO adsorption is less noticeable at lower temperatures. A 3D non-isothermal model was designed successfully, and Ansys Fluent software was used to validate the experimental results. The integrated designed configuration showed similar performance to that of the HT-PEMFC system fed with pure H_2_. Moreover, heat integration and heat efficiency were used from the electrochemical reaction to the MSR reaction.

Liu *et al.*^128^ developed a model designing a tubular HT-PEMFC integrated with a built-in packed bed reactor for the MSR reaction. The model was designed using COMSOL Multiphysics, including all the mass, heat and momentum equations as well as the MSR reaction, WGS reaction and MeOH cracking reaction, and was validated with experimental results showing good agreement. The main goal of the work was to assess the performance of the reaction and the thermal behaviour with various operating parameters. The results demonstrated that the reduction of voltage resulted in electrochemical heat production affecting the temperature distribution. Moreover, on the MeOH conversion, the voltage effect was studied for external potentials between 0.4 and 0.9 V, revealing that above 0.75 V there is no difference in the MeOH conversion values attributed to the low electrochemical heat production. For voltage values of 0.5 and 0.55 V, the MeOH reforming rate was found to be enhanced compared to that of the isothermal conditions achieving around 98% and 94%, respectively. Thus, the electrochemical heat can satisfy the required heat for the MSR reaction.

Chen *et al.*^129^ designed a 0D model including both MSR and HT-PEMFC technologies and studied the influence of different parameters such as the S/C ratio, reaction temperature, fuel cell number, MeOH catalytic combustion ratio and anode stoichiometry on the system efficiency and performance. As stated by this and many other studies, great effort must be put into enhancing the power generation. The optimisation of the S/C ratio should be made considering the CO selectivity, revealing that the S/C ratio of 1 had the highest CO selectivity. Maximum output power was obtained at 513 K with the efficiency increasing up to 37% compared to other reaction temperatures. Additionally, the anode reduction stoichiometry led to efficient electricity generation of almost 12%. The integrated system was able to sustain the heat generation from the anode.

A kinetic analysis of the MSR reaction on a commercial Cu/ZnO/Al_2_O_3_ catalyst and a theoretical study concerning the integration of the MSR reaction and HT-PEMFC were conducted by Ozcan and Akin,^130^ as shown in Fig. 8b. The system was developed for portable devices with power less than 15 W under optimum conditions. Using all the reaction rates and kinetics obtained from the analysis, a comparison was carried out between the obtained experimental and simulated conversion rates, showing good agreement between the results. The results from investigating different operating conditions in order to optimise the net power output of the integrated system were evaluated revealing that the system could generate 15.4 W power output, which is about twice that of the total required power (6.2 W).

A novel approach for an integrated system was suggested by Li *et al.*,^131^ suggesting the utilisation of heat waste from the HT-PEMFC system to supply the MSR reaction for H_2_ generation. An organic ranking cycle (ORC) was proposed as well, to recover the remaining heat for electrical power generation (Fig. 8c). A thermodynamic model was designed, and the results showed that the reaction rate was enhanced by the increase of temperature and the H_2_O/MeOH ratio, achieving a H_2_ yield above 95%. In addition, the implementation of the ORC subsystem using R245fa as the organic working fluid for waste heat recovery showed that the system has a better performance with the highest net output power of 1.64 W and efficiency of 17.65%. It was observed that higher anode pressures and hence lower cathode pressures could potentially improve the thermodynamic performance, minimising the cost and environmental pollution. The system optimisation resulted in an increase of net power output by around 48%.

Liang *et al.*^132^ incorporated a waste heat system that could be used in combination with cooling, heating and power generation for the integrated MSR-PEMFC system. An equation-oriented framework for the optimisation of the combined cooling, heating and power generation was proposed. The framework included the modelling of the MSR reaction and PEMFC system in detail, cooling and heating systems, heat integration, heat exchangers and energy and economic performance evaluation. It showed that the combined heating, cooling and power system was economically and thermodynamically advantageous to heat recovery. Moreover, the combination system showed 4.5% levelised cost of electricity of 0.2374 $ per kW per h compared with the conventional combination system. The heat exchanger network consists only about 2% of the total investment being effective in reducing the computational complexity.

## 6. Challenges & prospects

There is growing interest in the MSR reaction and more generally to MeOH for its importance as a chemical, fuel and feedstock. MeOH is one of the key H_2_-carrier molecules, with potential applications in the transportation sector. However, the technology faces several challenges, particularly in catalyst development. Effective catalysts must inhibit CO formation while increasing selectivity towards H_2_ and CO_2_. This is crucial not only to prevent catalyst deactivation but also to avoid poisoning the anode in PEMFCs. The design of such catalysts should focus on optimizing the performance at lower temperatures to suppress the RWGS reaction, thereby minimizing CO generation. If CO production cannot be completely suppressed, catalysts must be designed with materials that offer resistance to coking, tolerance to poisoning, and stability against sintering, thus extending their operational lifespan. Another key challenge is the high cost of catalyst materials. For example, although Pd-based catalysts have demonstrated exceptional conversion rates and H_2_ yields, their high cost hinders widespread adoption in large-scale applications.

A deeper understanding of the MSR reaction mechanism, metal–support interactions, surface adsorption energy, reaction pathways, and energy barriers is essential for developing effective catalysts. Many Cu-based catalysts have been thoroughly studied in terms of their reaction mechanisms, and there has been recent progress with Ni-based catalysts as well. Research on new materials for the MSR reaction should be conducted strategically, aiming to overcome CO formation and catalyst deactivation through mechanistic studies and the design of cost-effective catalysts for practical applications. Another critical aspect of catalyst design is the pore structure, which can often be random and result in the deactivation of catalytic sites. Proper design of the porous structure, accounting for heat and mass transfer phenomena, can maximize active reaction sites and improve the overall catalyst efficiency.

The selection of operational units is crucial for the traditional packed bed reactor to suffer from hot spots due to the thermal non-uniformity and the large pressure drop over the reactor's length. The reactor and herein the system concept should be able to carry out the MSR reaction in small- and large-scale applications and with high efficiencies. The MRs and micro-reactors are more advantageous compared to the conventional unit. MRs offer the selective separation of component species and could be employed directly to PEMFC units without any purification process, while the micro-reactors can overcome the pressure drop issues of packed bed reactors offering better heat and mass transfer rates. Nevertheless, the Pd membranes that have shown superior separation performance are limited by their enormous cost. Many efforts should be made in order to create cost-effective MRs and microreactors in terms of membrane materials and improve their scalability and operation in order to overcome potential issues.

PEMFC applications in the transportation sector were found to be a promising solution against the conventional fuel combustion engines. The direct utilisation of MeOH in PEMFC systems lacks performance due to the oxidation and poisoning of the anode. However, the transformation of the H_2_-energy carrier, MeOH, through the reforming reaction into H_2_ is more attractive due to the lower energy density. The main challenge is the heat recovery to maximise the overall efficiency and not waste the released heat from the HT-PEMFC systems. Many investigations suggest the heat integration between the reformer unit and HT-PEMFC to operate at similar temperatures. Another important parameter is the lifetime of the integrated system which most of the times depends on the stability of HT-PEMFC stacks. The optimisation of existing components used and the development of new ones are considered major contributors. Moreover, the lifetime of HT-PEMFC stacks in real-world applications is even more affected compared to the lab-scale experiments as there are many variations in the operating conditions, issues regarding the heat management, cell imbalances and failures. PEMFC systems also face other serious problems such as chemical, mechanical and thermal degradation and agglomeration. The main reason for this effect is the high operating temperatures, the acidic environment and the low humidity, which should be addressed soon in order to facilitate this technology transfer.

## Data availability

No primary research results, software or code has been included and no new data were generated or analysed as part of this review.

## Author contributions

Eleana Harkou: writing – original draft, review and editing. Hui Wang: writing – original draft, review and editing. George Manos: review, editing and supervision. Achilleas Constantinou: review, editing and supervision. Junwang Tang: review, editing and supervision.

## Conflicts of interest

There are no conflicts to declare.
